# Nitriding of Commercially Pure (CP) Titanium Grade 2 by the Active Screen Method

**DOI:** 10.3390/ma18204735

**Published:** 2025-10-16

**Authors:** Tadeusz Frączek, Milena Pilarska, Zbigniew Skuza, Rafał Prusak

**Affiliations:** 1 Department of Materials Engineering, Faculty of Production Engineering and Materials Technology, Czestochowa University of Technology, 42-201 Czestochowa, Poland; tadeusz.fraczek@pcz.pl; 2 BEKO Poland Manufacturing, 97-500 Radomsko, Poland; 3 Department of Production, Faculty of Production Engineering and Materials Technology, Czestochowa University of Technology, 42-201 Czestochowa, Poland; zbigniew.skuza@pcz.pl

**Keywords:** active screen, glow-discharge nitriding, commercially pure (CP) titanium

## Abstract

This paper addresses the formation of nitrided layers on CP Titanium Grade 2 substrates under direct current (DC) glow discharge conditions. In the preliminary research, the parameters for glow discharge plasma nitriding were selected, and the threshold process temperature was determined to ensure the formation of a continuous and uniform titanium nitride layer on the nitrided samples using the active screen method. On the other hand, the core research involved further detailed analysis of the formed surface layers at nitriding temperatures of 685 °C, 700 °C and 715 °C. It has been demonstrated that the nitriding of CP Titanium Grade 2 under DC glow discharge conditions using an active screen enables the formation of surface layers with uniform thicknesses and proper structures over the entire surface of the nitrided component.

## 1. Introduction

Plasma-assisted nitriding is widely used for surface hardening of components made not only from ferrous alloys, but also from an increasingly broad range of non-ferrous metals and alloys. The use of plasma as a crystallisation medium for deposited materials and an environment that directly interacts with the substrate surface is a fundamental attribute of modern surface treatment methods [1]. The concept of plasma was first introduced by Irving Langmuir in 1923. Glow discharge nitriding is a thermochemical treatment process that uses plasma to enrich the surface of the treated component with nitrogen. The nitriding process is usually carried out in a low-pressure, non-equilibrium, non-isothermal plasma environment with reactive gases at pressures ranging from 100 to 300 Pa. Low-pressure plasma strongly interacts with the surface of a solid, which is related to the kinetic energy and reactivity of plasma components, as well as the electrical charge accumulation and dissipation from the surface of the treated component.

### 1.1. Ion Nitriding of Titanium

J. M. Łachtin and J. D. Kogan [2] analyzed the rate of nitrided layer formation on titanium under direct current glow discharge conditions. The authors found that glow discharge enables obtaining an increased concentration of nitrogen on the cathode surface and an increase in the density of dislocations and vacancies in the near-surface region as a result of ion bombardment.

According to the aforementioned authors, those factors increase the nitrogen diffusion coefficient in titanium. Studies by H. Michael and M. Ganotis [3] have shown that permanent surface depassivation, nitrogen implantation, and, in all likelihood, the formation of TiN nitride in the atmosphere and its deposition on the cathode contribute to faster layer growth in the ion nitriding method than in other conventional methods controlled by nitrogen adsorption processes. H. Bollinger and associates [4] assumed that the model developed by K. Keller [5] for iron nitriding is also adequate for titanium. N^+^ nitrogen ions striking the cathode cause sputtering of titanium atoms, which, on contact with nitrogen atoms, form TiN microparticles. In subsequent collisions with the gas, these microparticles are deposited on the surface of the cathode—and thus on the surface of the treated component—and constitute a source of nitrogen diffusing into the material. M. Konuma and O. Matsumoto [6,7], considering the nitriding of titanium in nitrogen as well as in nitrogen and hydrogen using a radio-frequency glow discharge, concluded that the phenomena controlling the layer growth process are ion adsorption and nitrogen diffusion. Furthermore, according to Matsumoto [7], the beneficial effect of hydrogen addition on the layer growth lies in the reduction in cathodic sputtering. M. B. Liu et al. [8] distinguished two stages of ion nitriding. The first involves the implantation of nitrogen ions and sputtering of titanium atoms and depositing them on the cathode in the form of TiN. The second, involving the formation of permanent nitride layer on the surface, leads to thermochemical equilibrium and in-depth nitrogen diffusion. H. K. Hu et al. [9,10], while investigating the interaction of N_2_^+^ nitrogen ions with the surface of a solid (Re, Ti), developed a reaction model for the formation of a new phase, which, for titanium, is as follows: N_2_^+^ and N^+^ ions undergo neutralization at a distance of 0.5–0.6 nm from the solid surface; neutral, high-energy N_2_^+^ particles dissociate in the collision with the surface if their energy is greater than 1.52 × 10^−18^ J; and the nitrogen atoms (N), through successive collisions with titanium atoms, lose energy and form stable titanium nitride (TiN).

The papers discussed mostly exposed the role of ions in the interaction of ionized nitrogen with the surface of titanium, neglecting the role of adsorption processes [11].

Zhecheva [12] presented a simplified model for the formation and growth of individual nitrided layers on commercially pure titanium during ion treatment below the allotropic transformation temperature of α → β (862 °C). The model assumes that the ion nitriding process first leads to the formation of a diffusion zone α(N)-Ti, also referred to in the literature as Tiα(N). Once the nitrogen concentration resulting from the solubility of this element in the α phase is exceeded, TiN and Ti_2_N nitride zones are formed. The Ti_2_N and TiN sublayers form a nitride layer, which is separated from the Tiα phase with a Tiα(N) diffusion layer [12,13,14]. Phase transformations on the surface of the nitrided component can be expressed as follows: α(Ti) → α(N) → Ti_2_N → TiN. In the nitriding of titanium alloys, it is necessary to consider the influence of alloying elements on the formation of titanium nitrides, as well as on the formation of stable and metastable alloying element nitrides [12].

A different view was presented by the author of another paper [15]. In the proposed model of glow discharge nitriding of commercially pure titanium, using an active screen, it has been assumed that the TiN nitride formed in the initial stage of the nitriding process is transformed into Ti_2_N nitride and free nitrogen diffusing into the substrate material. Further into the process, the Ti_2_N nitride is decomposed into titanium and nitrogen diffusing deep into the nitrided substrate. An important aspect of the research was to demonstrate that ion nitriding on a cathode, using an active screen already at 530 °C and over a long-term process of 17 h, leads to the formation of a nitrided layer on the surface of the sample directly adjacent to the cathode. It should be noted, however, that the author of this paper did not determine whether the thicknesses of the nitrided layers formed on the top surface and bottom of the sample are comparable. The literature data indicate the possibility of forming a nitrided layer on such a surface only at a much higher temperature (around 800 °C) (but without an active screen).

In [16], the authors show that, at cathode nitriding temperatures below 580 °C, a nitrided layer on titanium, like on iron, forms only on surfaces surrounded by glow discharge plasma. This is because, below this temperature, titanium nitride can only form by reactions involving atomic nitrogen. At temperatures above 580 °C, on the other hand, a nitrided layer also forms on surfaces shielded from the glow discharge, as a result of the reaction of titanium with molecular nitrogen, which can be facilitated by the use of an active screen.

The above statement is not very precise, so it is worth noting that the nitrogen molecule undergoes dissociative chemisorption and, therefore, atomic nitrogen participates in the synthesis of titanium nitride.

### 1.2. Ion Nitriding Using an Active Screen

The active screen plasma nitriding technology was introduced and patented in 1999 by J. Georges [17]. In the global literature, the aforementioned nitriding variant is also referred to as trough cage or cathodic cage [18,19,20,21].

The use of an active screen in the ion nitriding process makes it possible to limit the occurrence of unfavorable phenomena observed during cathodic nitriding, i.e., the cavity cathode effect, the effect of material volumes with different geometries on temperature distribution, and the edge effect caused by the build-up of electrical charge at the edges of the treated component [18,22,23].

In the active screen nitriding (ASN) technology, the treated component is placed on an isolated substrate under a perforated element (screen, mesh) polarised by cathodic voltage. The presented variant differs significantly from cathodic nitriding, because, due to the absence of direct polarisation, ion bombardment of the sample surface does not occur. Instead, nitrogen-carrying particles sputtered from the surface of the active screen reach and react with the surface of the treated component. When a new, previously unused screen is used for nitriding, the atoms of the constituent metal elements are initially sputtered from its surface. After a certain period of the process, once the screen becomes nitrided or nitride phases have deposited on its surface, the sputtered species consist predominantly of nitride phases [24].

The use of an active screen is also possible for other variants of ion nitriding, in which the differences in the obtained surface layers result from differences in the polarisation of the treated component, cathodic voltage, anodic voltage, or complementary potential (additional polarization potential) [15,25]. In the case of cathodic nitriding with an active screen, the layers formed are characterised by a greater thickness of nitrided layers compared to the process without an active screen [15]. However, in such a case, the components exhibit technological defects (e.g., edge effect), which are eliminated when nitriding is performed solely at the plasma potential.

Also, in nitriding processes using a complementary potential with an active screen, the layers formed are characterised by greater thicknesses than those obtained in processes without an active screen [15].

From a technological perspective, in addition to the absence of the edge effect, ion nitriding using an active screen eliminates the occurrence of a temperature gradient that can lead to uneven nitriding of complex-shaped components, as confirmed by tests on samples of various sizes. This ensures uniformity of the nitriding process across surfaces of components differing in geometry and dimensions, enabling consistent and repeatable results [26]. Polarisation of the active screen generates additional voltage pulses, the value of which is several times higher than the voltages during nitriding on the cathode (without the active screen). As a result, ions and other active plasma species achieve high velocities and, consequently, high kinetic energy of the particles (several hundred electron volts). Those processes lead to the implantation of active plasma species into the surface of the nitrided component, creating a non-equilibrium nitrogen-saturated zone, which facilitates nitrogen diffusion deep into the substrate [15].

The dissimilarity of the nitriding mechanism using the active screens compared to classical cathodic nitriding has been confirmed by spectroscopic (OES) studies. Spectral analysis of the glow discharge under the screen showed the occurrence of spectral lines originating from N^+^ nitrogen ions, N atomic nitrogen and Hα hydrogen, which demonstrates a significant contribution of these plasma species under the screen. More detailed studies have additionally shown the presence of a small amount of N_2_ molecular nitrogen [27]. Noteworthy is the absence of Fe-derived spectral lines and NH radical bands. This may be due to their low concentration in the plasma below the screen.

Currently accepted models for titanium nitriding in glow discharge plasma using an active screen are still being verified and improved. Due to the various process variants used, variable parameters and types of plasma generated, the existing nitriding models are not unambiguous. The ambiguity of the model is even greater in the case of ion nitriding using an active screen [27,28,29].

The development of a comprehensive description of nitriding mechanisms involving a plasma environment is complex and requires the consideration of a wide variety of phenomena, i.e., shaping the characteristics of the discharge and the physical and chemical state of the plasma, the course of nitrogen transport processes from the plasma area to the substrate, the interaction of the active particles with the surface of the nitrided component, and the course of nitrogen transport processes deep into the nitride substrate [1].

The practical aim of this study is to determine the threshold nitriding temperature at which a homogeneous surface layer is formed on the entire surface of nitrided commercially pure titanium Grade 2 during active screen plasma nitriding. The formation of titanium nitrides on the underside of the sample in processes without the application of an active screen is possible, but only at high temperatures. Previous work by the authors has shown that, in processes with active shielding, titanium nitrides can also form on the underside of the sample (i.e., the surface directly adjacent to the cathode) already at 530 °C. Notably, however, the layers produced in this way were not continuous, and the process time was as long as 17 h [15]. The main objective of the research, the results of which are presented in this article, was to determine the minimum temperature at which titanium nitride layers of comparable thickness are formed on the whole surface of the sample (including the surface directly adjacent to the cathode). For economic reasons, a process time of 5 h was chosen as a boundary condition. This time corresponds to that of the shortest processes used in industrial practice. Research of this kind, using the indicated process parameters, has never been conducted before.

## 2. Materials and Methods

**Figure 1:**
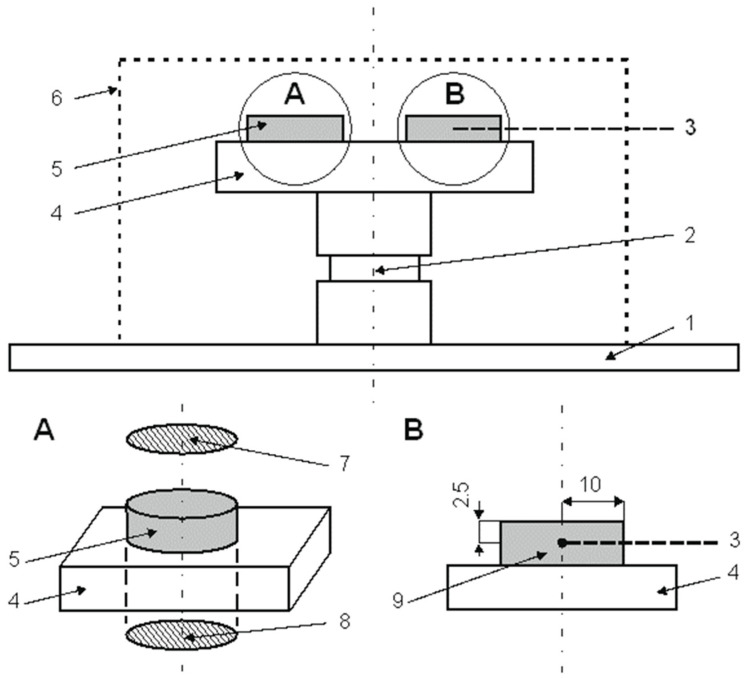
Graphical diagram of a commercially pure titanium Grade 2 sample on the cathode, 1—cathode; 2—titanium roller; 3—temperature sensor (TP-203K-1b-600-1,5 (Conrad Electronic SE, Hirschau, Deutschland)); 4—titanium table; 5—sample; 6—active screen; 7—surface A; 8—surface B; 9—control sample.

Glow discharge plasma nitriding processes were carried out on commercially pure titanium Grade 2 according to ASTM (West Conshohocken, PA, USA)—UNS R50400 [30]. In order to intensify the ion nitriding process, a cylindrical active screen made of perforated Grade 2 titanium sheet was placed on the cathode. To define the effect of bombarding the nitrided substrate with nitrogen ions and other active plasma species, the samples were placed on a flat cathode according to the graphical diagram shown in Figure 1.

Before each glow discharge plasma nitriding process, the surface of the cathode was carefully polished using 220-grit sandpaper. This procedure was intended to ensure the repeatability of the process and to minimise the effect of cathode roughness on selected properties of the surface layers formed on the bottom surface of the sample during nitriding.

According to literature data [11], surface A was exposed to bombardment with ions, neutral particles and other active species generated in the glow discharge plasma, while the surface layers on surface B were likely formed as a result of classical gas nitriding (glow discharge does not occur in such a narrow space as is formed between the sample and the cathode).

Based on the literature analysis concerning the nitriding of commercially pure titanium and its alloys, the parameters for the preliminary and main nitriding processes have been established and are summarised in Table 1.

To eliminate the influence of alloying elements on the properties of the obtained surface layers, glow discharge plasma nitriding processes were carried out on technically pure Ti99.2 titanium compliant with EN10204-3.1 (Grade 2 according to ASTM [30]) with the chemical composition shown in Table 2.

It was assumed that nitriding of commercially pure titanium according to the Ti-N equilibrium system would form a layer of titanium nitrides (TiN, Ti_2_N) and a nitrogen-enriched Tiα(N) solid solution on the surface of the treated component.

Given the strong affinity of titanium for oxygen, which results in the formation of a dense surface layer of titanium oxides (rutile-TiO_2_) preventing the nitriding process from proceeding, a sputtering atmosphere consisting of hydrogen and argon (H_2_ 67% + Ar 33%) was used to activate the surface and remove the oxides [15]. The argon used in the sputtering process has a relatively high mass compared to other gases used in glow discharge treatments, which allows for its ions to acquire a relatively high kinetic energy to sputter the cathode surface and the nitrided component by ion bombardment. In addition, it should be noted that argon, being a noble gas, does not chemically react with the treated surface. Although hydrogen is commonly included in nitriding atmospheres, due to the potential for hydrogen-induced embrittlement of titanium reported in the literature [11], in this study, a pure nitriding atmosphere consisting of 100% N_2_ by volume. The temperature of the nitriding process was controlled by adjusting the power of the electric current generating the glow discharge, and monitored using a control sample placed on the cathode, inside of which a type K thermocouple was installed.

The prepared transverse metallographic sections were subjected to, the following:

- Observations using a Carl Zeiss Axiovert 25 light microscope (Carl Zeiss, Oberkochen, Deutschland) and a Jeol 6610Lv scanning electron microscope (JEOL Ltd, Tokio, Japan);
- Examination of the elemental distribution profile using a LECO’s GDS 850A spectrometer (GDOES—Glow Discharge Optical Emission Spectroscopy) (LECO Corporation, St. Joseph, MI, USA) equipped with a Grimm discharge lamp (LECO Corporation, St. Joseph, MI, USA) with a cathode diameter of Ø 4 mm. The spectrometer utilizes the technique of glow discharge optical emission spectrometry in a vacuum. During analysis, the sample surface was bombarded with a stream of ionized argon, resulting in the uniform sputtering of successive layers of atoms from the sample surface. This process was conducted at reduced pressure and without additional thermal effects on the sample surface;
- Examination of surface microhardness of the material in the as-delivered condition and after ion nitriding processes using FutureTech FM-7 microhardness tester (Future-Tech Corporation, Kawaguchi, Japan) Knoop method (providing higher measurement accuracy compared to the Vickers method due to the longer diagonal of the indentation, recommended for measuring the microhardness of thin surface layers of hard and brittle materials without risk of indenter damage).

Since identification of surface layers with thicknesses ranging from 0.1 μm to 4 μm under an optical microscope is problematic and often prone to error, it is useful to employ the diagonal grinding method, which physically enlarges the analysed area. This method is not widely used because of the labour intensity due to the need for precise preparation. The use of a large-diameter metallographic specimen in the form of a sphere is easier and more suitable for thickness measurements and especially for the analysis of the structure of thin surface layers. The metallographic specimen has the characteristics of oblique grinding and physically extends the area of the layer to be analysed. The slight sphericity of the grind does not affect the quality of microscopic observations. The Sphere Tester stand for metallographic analysis of thin films and coatings using spherical grinding was designed and built at the Institute of Precision Mechanics in Warsaw. The thickness of the layer revealed by the examination was calculated based on Formulas (1) and (2).

(1) $$g=\frac{1}{2}\left(\sqrt{4R^{2}-d^{2}}-\sqrt{{4R}^{2}-D^{2}}\right)$$

(2) $$g=\frac{x*y}{2R}$$

where

R—radius of the sphere;

D—abrasion diameter;

d—abrasion diameter of the exposed substrate;

y—width of the exposed layer on the spherical specimen;

x—abrasion diameter reduced by the thickness of the exposed layer (x = D − y).

## 3. Results

### 3.1. Preliminary Research Results

**Figure 2:**
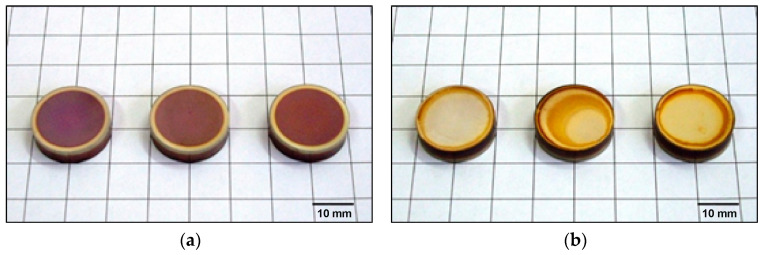
Macrophotographs of samples after ion nitriding at 685 °C, 700 °C and 715 °C: (**a**) surface A, (**b**) surface B.

Macroscopic observations showed that the surfaces of the samples exposed to the glow discharge plasma are characterised by a brown colour (Figure 2), which may indicate the presence of oxygen during the nitriding. This clearly suggests insufficient purging of the furnace atmosphere prior to the nitriding process. The occurrence of the edge effect was also observed, which, according to the literature, arises as a result of excessive accumulation of electrical charges on the sharp edges of the nitrided component. The surfaces in direct contact with the cathode had a different colour; furthermore, a TiN layer was also observed, as evidenced by the golden colouring of the sample surfaces.

**Figure 3:**
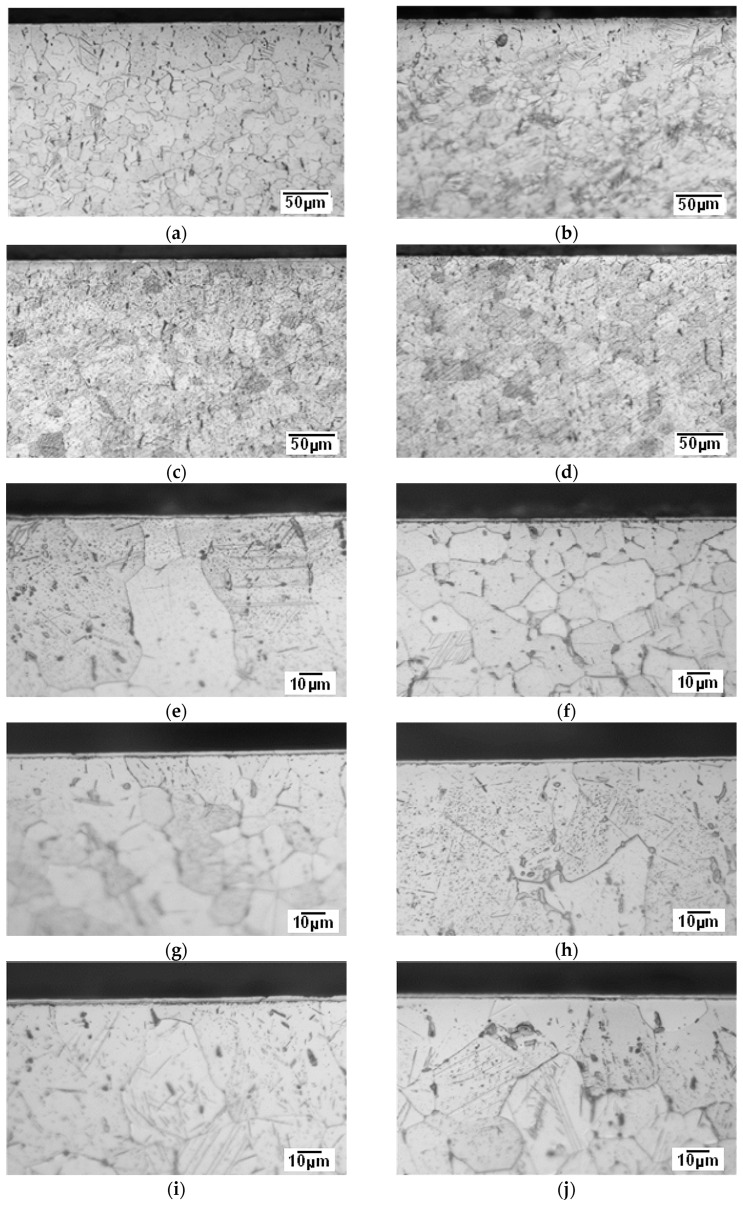
Microstructure of surface layers on commercially pure titanium Grade 2 substrates after ion nitriding; light microscopy at temperatures: 655 °C—(**a**) surface A, (**b**) surface B; 670 °C—(**c**) surface A, (**d**) surface B; 685 °C—(**e**) surface A, (**f**) surface B; 700 °C—(**g**) surface A, (**h**) surface B; 715 °C—(**i**) surface A, (**j**) surface B.

Analysis of the obtained microstructures (Figure 3) showed that the glow discharge plasma nitriding processes, under the adopted parameters (Table 1), ensure the formation of homogeneous surface layers over the entire surface of the nitrided sample at a threshold temperature of 700 °C.

**Figure 4:**
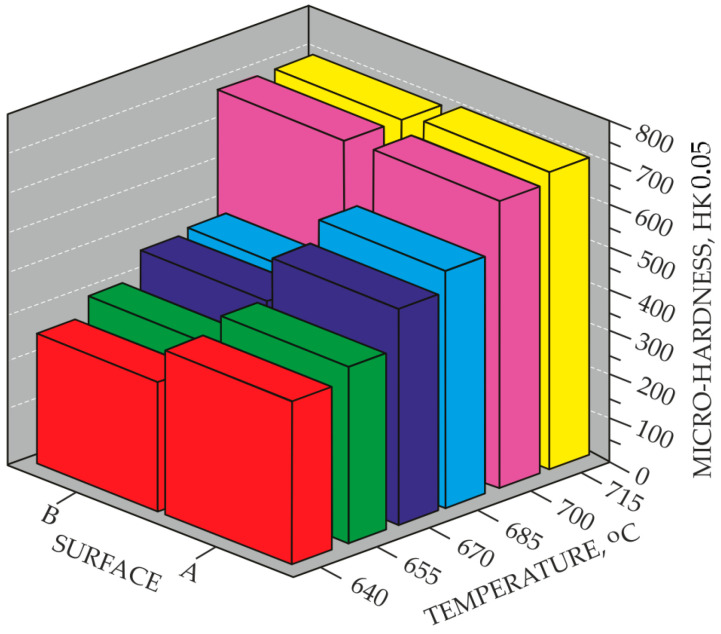
Surface microhardness of the top layers after the conducted nitriding processes.

Processes carried out at lower temperatures, i.e., below 700 °C, result in a reduction in the quality of the nitrided layers, as evidenced by the absence of a continuous titanium nitride layer, particularly on surface B. The above statements are confirmed by the microhardness measurements (Figure 4). Samples nitrided at temperatures below 700 °C exhibited significant differences in microhardness on individual surfaces. For example, the difference in hardness between surface A and surface B of a sample nitrided at 685 °C is 190 HK0.05. In contrast, nitriding at 700 °C ensured similar microhardness of the tested areological system on both surfaces, i.e., 705 HK0.05 on surface A and 687 HK0.05 on surface B, respectively.

The results of preliminary tests were illustrative; therefore, the main tests involved a detailed analysis of the surface layers obtained according to the adopted testing methodology.

### 3.2. Main Research

Based on observations using scanning microscopy and analysis of elemental distribution via GDOES, the structure of the surface layers formed on commercially pure titanium Grade 2 substrate was characterised. The analysis of both surface A and surface B of the nitrided sample allowed for the assessment of the effect of the glow discharge plasma on the diffusion depth of nitrogen and other analysed elements (titanium, carbon, oxygen). The results are presented graphically as quantitative depth profiles of the individual elements as a function of distance from the surface.

**Figure 5:**
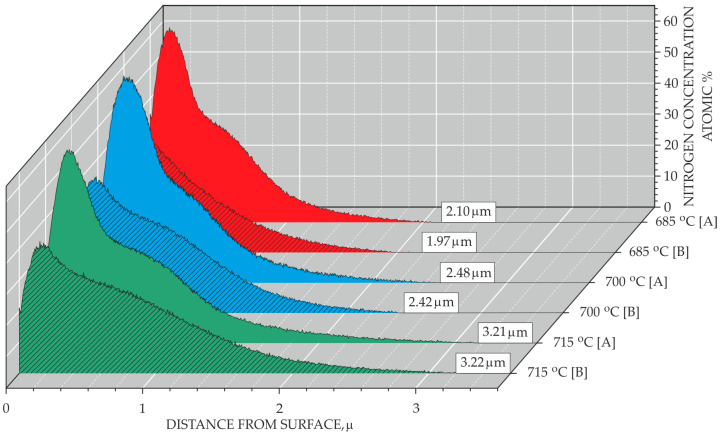
Depth profile of nitrogen in the surface layers as a function of the nitriding process temperature.

Figure 5 shows the depth profiles of nitrogen distribution in the surface layers after ion nitriding treatment. For surface layers with a low nitrogen diffusion depth produced with the adopted treatment parameters, the determination of this parameter by other methods (e.g., metallographic methods) is problematic or impossible. The recorded curves have a typical course for layers of a diffusive nature, i.e., a decrease in nitrogen concentration was observed with increasing distance from the nitrided surface. In addition, an increase in the nitrogen diffusion depth was observed as the temperature of the nitriding process increased. For the treatment at 715 °C, the same nitrogen diffusion depth was found on both surface A and surface B.

**Figure 6:**
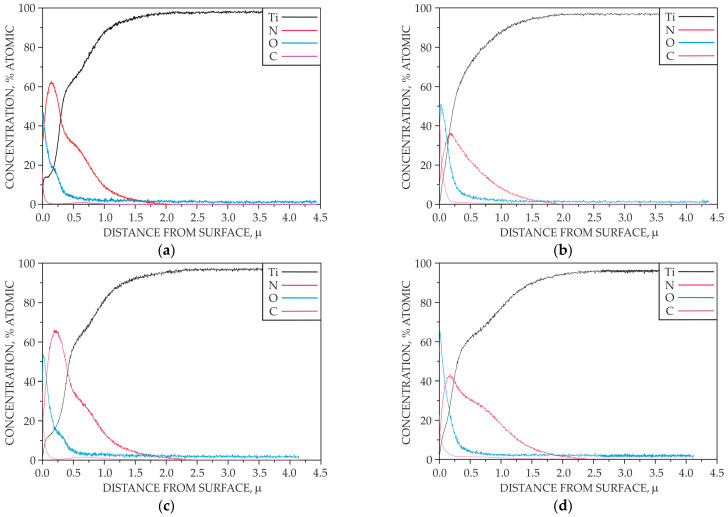

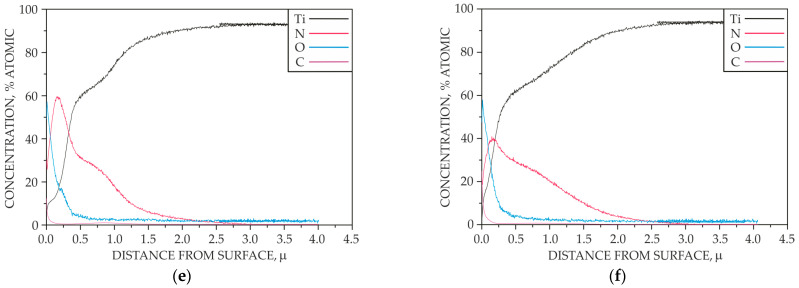
Depth profile of the analyzed elements in the surface layer at temperature: 685 °C (surface A—(**a**), surface B—(**b**)), 700 °C (surface A—(**c**), surface B—(**d**)), 715 °C (surface A—(**e**), surface B—(**f**)).

Figure 6 shows the concentration distribution of selected elements in the surface layers after the ion nitriding treatment.

It has been shown that the oxygen and carbon content in the surface layers produced on surface B is higher than that of the layers exposed to the glow discharge plasma. This fact is mainly due to the desorption of particles present on the cathode, as well as contamination radicals from residual gases in the vacuum apparatus. On the other hand, ion collisions with the cathode cause cathodic sputtering of oxygen, carbon and nitrogen atoms chemically bonded to titanium. It is noteworthy that the presence of oxygen is limited to the strictly near-surface zone, both in the surface layer on surface A and surface B. The composition of the reactive atmosphere plays a crucial role in the nitriding of titanium. Titanium exhibits a strong tendency toward passivation; therefore, the nitriding atmospheres used should be free of contaminants. The oxygen and carbon compounds present in the reactive atmosphere, even in trace amounts, may participate in the growth of the nitrided layer on the titanium substrate, adversely affecting its properties. It is worth mentioning that the presence of oxygen can also be caused by leaks in the vacuum apparatus. Carbon in the reactive atmosphere mainly originates from oil vapours emitted by the vacuum pump and should, therefore, be regarded as an unavoidable impurity, the effect of which is reduced by the glow discharge plasma.

**Figure 7:**
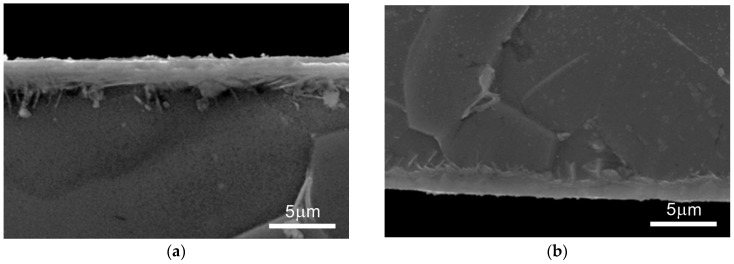

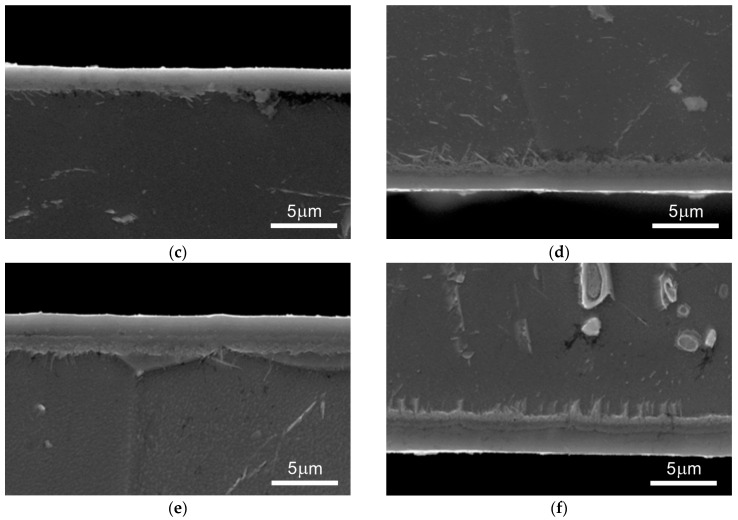
Microstructure of the surface layer on the commercially pure titanium Grade 2 substrate: scanning microscopy at temperatures 685 °C (surface A—(**a**), surface B—(**b**)), 700 °C (surface A—(**c**), surface B—(**d**)), 715 °C (surface A—(**e**), surface B—(**f**)).

Representative microstructural images show homogeneous nitrided layers with a characteristic zonal structure (Figure 7).

**Figure 8:**
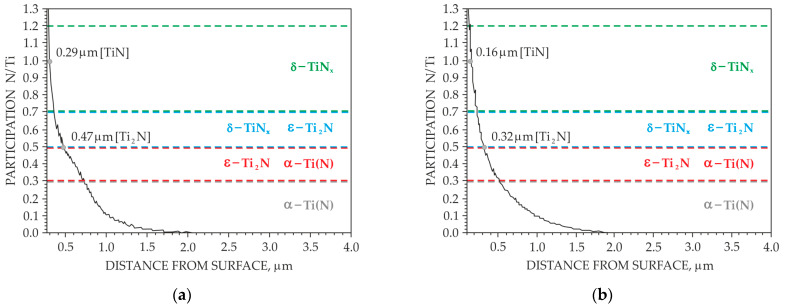

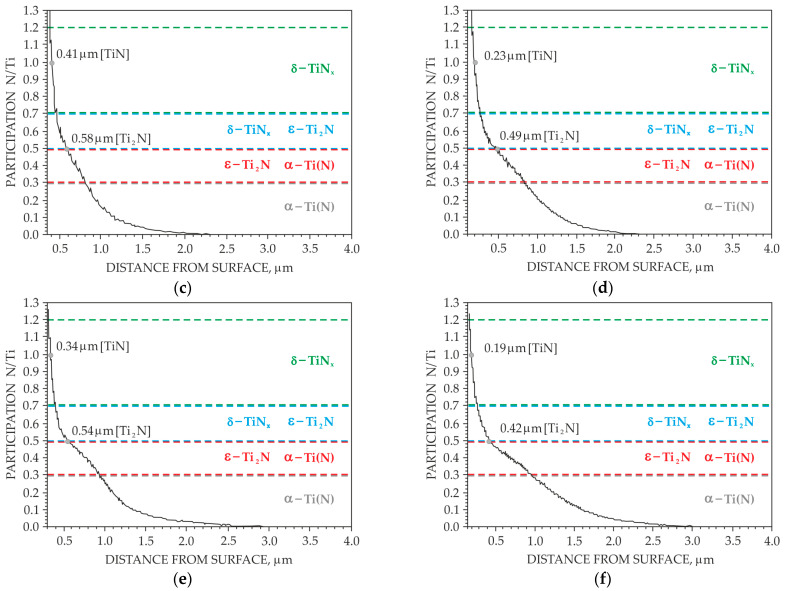
N/Ti concentration ratio for samples nitrided at 685 °C (surface A—(**a**), surface B—(**b**)), 700 °C (surface A—(**c**), surface B—(**d**)), 715 °C (surface A—(**e**), surface B—(**f**)).

It has been demonstrated that nitriding at 700 °C and 715 °C forms surface layers of similar thickness over the entire observed surface. In contrast, a significant irregularity in layer thickness is observed for samples nitrided at the lower temperature of 685 °C, particularly on surface B. Based on the distribution of nitrogen and titanium concentrations and their atomic ratios in the surface layers, a graphical diagram illustrating the distribution of individual nitride phases was proposed (Figure 8). In the region where the nitrogen-to-titanium atomic ratio (N/Ti) is assumed. For ratios between 0.3 and 0.5, both the below 0.3, the presence of a nitrogen solid solution in titanium is below 0.3, the presence of a nitrogen solid solution in titanium, Tiα(N), is assumed. For ratios between 0.3 and 0.5, both the Tiα(N) phase and the tetragonal ε-Ti_2_N phase are expected to occur. In the range of 0.5 to 0.7, both the ε-Ti_2_N phase and the cubic δ-TiN phase are observed.

## 4. Discussion

Based on preliminary macroscopic observations of the nitrided samples (Figure 2), it was found that the surfaces subjected to the glow discharge plasma are characterised by a brown colour, which may be evidenced by the presence of oxygen. This clearly suggests insufficient purging of the furnace atmosphere prior to the nitriding process. Surfaces B, on the other hand, are characterised by a golden colour, which confirms the presence of the TiN phase. Obtaining a golden colour for nitrided surfaces is currently one of the highly desirable features. This colour strongly depends on the chemical composition, texture and roughness of the surface layer.

Microstructural observations of the nitrided layers revealed that compact surface layers were formed on both surface A and surface B at the threshold temperature of 700 °C (Figure 3) under the applied nitriding parameters. These microstructural findings correlate well with the microhardness results (Figure 4), which clearly show that the microhardness values of both surface A and surface B are comparable at the above-mentioned threshold temperature.

It was confirmed, in agreement with the literature data, that, at the threshold temperature of 700 °C for the ion nitriding process, uniform surface layers on the surfaces in direct contact with the cathode (surface B) are formed by adsorption processes involving atomic nitrogen.

In the main research, a detailed examination of the obtained surface layers was carried out. The research made it possible to determine the role of the glow discharge plasma in the nitriding of the commercially pure titanium Grade 2. The beneficial effect of ion bombardment and other active plasma species on the surface processes was confirmed, including a noticeable reduction in the oxygen and carbon content within the nitrided layer.

Based on GDOES analysis, it was found that, for the nitriding process carried out at a temperature of 700 °C, the nitrogen diffusion depth was 2.48 µm for surface A and 2.42 µm for surface B (Figure 5). The ion nitriding process conducted at 715 °C resulted in the same nitrogen diffusion depth for both surfaces A and B of the nitrided specimen, i.e., 3,22 µm. The obtained layers are thicker than in PVD coatings, which is an expected result consistent with the research of other authors [31,32]. It should be expected that extending the process time would contribute to an increase in the thickness of the obtained layers, even when using lower temperatures. Studies by other authors [33,34,35] led, among other things, to obtaining a thickness of 6 µm in processes carried out at a temperature of 600 °C, with the process duration being much longer and amounting to 20 h. The thickness of the layers can also be increased by introducing into the atmosphere H_2_ and the N-rich top layer of TiN layer with coarse grain structure resulted in the combined effect of the introduction of H_2_ and the plasma action [36]. The thickness of the obtained layers could also be increased by modifying the bias voltage; research by other authors [35] indicates that the change in bias voltage has a significant impact on the obtained layer thicknesses.

Additionally, the depth profiles confirmed a higher content of oxygen and carbon on the surfaces of the samples where only adsorption phenomena could occur, i.e., on surface B (Figure 6, Figure 7 and Figure 8).

An analysis of the micrographs of the produced surface layers revealed that nitriding at 700 °C and 715 °C ensured the formation of surface layers of comparable thickness across the entire observed area (Figure 7a–f). In contrast, a high irregularity in the thickness of the surface layers was observed for the nitriding process carried out at a lower temperature, i.e., 685 °C, particularly on surface B (Figure 7b).

Based on the distribution of nitrogen and titanium concentrations and their atomic ratios in the surface layers, a graphical diagram illustrating the occurrence of the individual nitride phases was proposed (Figure 8). It was assumed that in the region where the nitrogen-to-titanium atomic ratio (N/Ti) is below 0.3, a zone of solid solution grains of nitrogen in titanium Tiα(N) occurs. In the range from 0.3 to 0.5, both a Tiα(N) phase and a tetragonal ε-Ti_2_N phase were identified. For N/Ti ratios between 0.5 and 0.7, the presence of both ε-Ti_2_N and cubic δ-TiN phase was observed. An increase in the nitriding temperature not only leads to a greater thickness of the nitrided layer system, but also results in a change in the relative amounts of the TiN and Ti_2_N phases [10].

It should be noted that, in order to provide a comprehensive evaluation of the quality of the nitrided layers obtained under the adopted parameters, several additional studies were conducted, including X-ray phase analysis, surface topography, and adhesion tests. The authors of this paper intend to present the results of these tests in a subsequent publication.

## 5. Conclusions

This research fills a gap in knowledge related to the formation of nitride layers across the entire surface of a nitrided component. The minimum temperature for this process was determined at an optimum nitriding time of 5 h from an economic standpoint. The research and observations carried out give rise to the following observations and conclusions:

1. In order to eliminate the brown coloring of the surface, the adopted parameters of the ion nitriding treatment ensure the formation of homogeneous surface layers of a diffusive nature, over the entire surface of the nitrided sample, at a limit temperature of 700 °C. However, it was only for the treatment carried out at 715 °C that the same depth of nitrogen diffusion was found on both surface A and surface B.
2. It was found that homogeneous surface layers on the direct cathode contact surface (surface B) are formed at the ion nitriding process limit temperature of 700 °C by adsorption processes involving atomic nitrogen.
3. In order to eliminate the brown coloring of the surface of the nitrided samples, which is indicative of the presence of oxygen during nitriding, the flushing time of the furnace atmosphere must be increased before the main nitriding process.
4. The phenomenon of ion bombardment, as well as other active plasma components, contributes to the reduction of carbon and oxygen content in the produced surface layers on surface A of the nitrided sample.

## Figures and Tables

**Table 1:** Parameters of nitriding processes for commercially pure titanium Ti99.2.

Process ^1^	Temperature °C	Time h	Pressure Pa	Atomizing Atmosphere mL/min	Nitriding Atmosphere mL/min
1	640	5	150	360 H_2_, 160 Ar	500 N_2_
2	655	5	150	360 H_2_, 160 Ar	500 N_2_
3	670	5	150	360 H_2_, 160 Ar	500 N_2_
4	685	5	150	360 H_2_, 160 Ar	500 N_2_
5	700	5	150	360 H_2_, 160 Ar	500 N_2_
6	715	5	150	360 H_2_, 160 Ar	500 N_2_

^1^ Each process was carried out using an active screen.

**Table 2:** Chemical composition of technical titanium Ti99.2.

Element	C	Fe	O	N	H	Ti
Mass percentage	0.01	0.08	0.12	0.01	0.001	rest

Chemical composition in accordance with Daido Steel Ca Ltd. (Higashi-ku, Nagoya, Japan) certificate.

## Data Availability

The data can be accessed from Czestochowa University of Technology.
